# Physicians’ Attitudes Toward Prescribable mHealth Apps and Implications for Adoption in Germany: Mixed Methods Study

**DOI:** 10.2196/33012

**Published:** 2021-11-23

**Authors:** Florian Dahlhausen, Maximillian Zinner, Linn Bieske, Jan P Ehlers, Philip Boehme, Leonard Fehring

**Affiliations:** 1 Didactics and Educational Research in Health Care, Faculty of Health School of Medicine Witten/Herdecke University Witten Germany; 2 Faculty of Health School of Medicine Witten/Herdecke University Witten Germany

**Keywords:** mobile health, mHealth, digital health, apps, physicians, general practitioners, technology acceptance, adoption

## Abstract

**Background:**

In October 2020, Germany became the first country, worldwide, to approve certain mobile health (mHealth) apps, referred to as DiGA (Digitale Gesundheitsanwendungen, in German, meaning digital health applications), for prescription with costs covered by standard statutory health insurance. Yet, this option has only been used to a limited extent so far.

**Objective:**

The aim of this study was to investigate physicians’ and psychotherapists’ current attitudes toward mHealth apps, barriers to adoption, and potential remedies.

**Methods:**

We conducted a two-stage sequential mixed methods study. In phase one, semistructured interviews were conducted with physicians and psychotherapists for questionnaire design. In phase two, an online survey was conducted among general practitioners, physicians, and psychotherapists.

**Results:**

A total of 1308 survey responses by mostly outpatient-care general practitioners, physicians, and psychotherapists from across Germany who could prescribe DiGA were recorded, making this the largest study on mHealth prescriptions to date. A total of 62.1% (807/1299) of respondents supported the opportunity to prescribe DiGA. Improved adherence (997/1294, 77.0%), health literacy (842/1294, 65.1%), and disease management (783/1294, 60.5%) were most frequently seen as benefits of DiGA. However, only 30.3% (393/1299) of respondents planned to prescribe DiGA, varying greatly by medical specialty. Professionals are still facing substantial barriers, such as insufficient information (1135/1295, 87.6%), reimbursement for DiGA-related medical services (716/1299, 55.1%), medical evidence (712/1298, 54.9%), legal uncertainties (680/1299, 52.3%), and technological uncertainties (658/1299, 50.7%). To support professionals who are unsure of prescribing DiGA, extended information campaigns (1104/1297, 85.1%) as well as recommendations from medical associations (1041/1297, 80.3%) and medical colleagues (1024/1297, 79.0%) were seen as the most impactful remedies.

**Conclusions:**

To realize the benefits from DiGA through increased adoption, additional information sharing about DiGA from trusted bodies, reimbursement for DiGA-related medical services, and further medical evidence are recommended.

## Introduction

Health care systems worldwide are struggling with rising costs [1]. Great hopes are being pinned on digital health, such as mobile health (mHealth) apps, to address the root causes of these burdens [2]. mHealth apps are said to have great potential for improving health outcomes in numerous ways [3] (eg, increased health competence [4], better symptom management [5], and improved adherence to chronic disease management [6]). Despite these benefits, several factors are hindering widespread adoption of mHealth solutions, including technological, social, and organizational factors [7], limited reimbursement [8,9], and further need for empirical research on the effectiveness of mHealth [10].

To address some of these challenges, in October 2020, Germany became the first country, worldwide, to grant statutorily insured individuals an entitlement to use certain mHealth apps at the expense of health insurers [11]. These apps are referred to as DiGA (Digitale Gesundheitsanwendungen, in German, meaning digital health applications), a subset of the over 280,000 health, fitness, and medical apps available worldwide at the end of 2020 [12,13]. DiGA are medical devices primarily based on digital technologies that support the detection, monitoring, treatment, mitigation, or compensation of disease, injury, or disability. Additionally, they must have successfully cleared an assessment of positive care effects and product qualities—most importantly, safety and suitability for use, data protection and security, and interoperability—by BfArM (Bundesamt für Arzneimittel und Medizinprodukte, in German, meaning the German Federal Institute for Drugs and Medical Devices) [14]. All such apps would then be included in the official DiGA directory of prescribable, reimbursable apps.

As app reimbursement is only possible when prescribed by a physician or psychotherapist or when approval had been directed by the health insurer, health care professionals—especially in the outpatient care sector—play an important role in the implementation process [15]. Five months after their introduction, only 3700 DiGA had been prescribed and reimbursed, increasing to 17,000 DiGA by 10 months after their introduction [16,17].

Vast research has investigated the technological, structural, and human factors that may influence technology adoption by health care professionals [18], most prominently through innovation adoption and diffusion theories by Rogers [19], the technology acceptance model [20], and the unified technology acceptance and use of technology theory [21]. What followed was empirical work introducing various country-specific surveys on health care professionals’ mHealth adoption [22-24] as well as studies focused on specific medical disciplines and technologies, ranging from telemedicine and remote monitoring [25,26] to medical app use [27].

To our knowledge, no study has systematically examined adoption of mHealth apps by physicians and psychotherapists in the outpatient care sector—referred to as health care professionals in the following sections—in the context of institutionalized programs with reimbursement of government-certified, prescribable apps, as is the case with DiGA in Germany. This study aims to fill this gap by analyzing health care professionals’ attitudes and prescription intentions toward DiGA, as well as barriers to adoption and potential remedies. It includes findings from the largest survey on mHealth adoption by health care professionals in Germany. Given Germany’s unique and leading approach to mHealth app adoption, the findings can be applied to other countries looking to expand access to mHealth apps.

## Methods

We used a mixed methods approach consisting of semistructured interviews followed by an online survey, which was developed based on the findings of the initial qualitative interviews.

### Exploratory Interviews for Survey Questionnaire Design

We first conducted a structured literature review of both existing technology adoption literature and global case studies. Drawing on these bodies of literature, we developed a semistructured interview guide for interviews with physicians and psychotherapists about their views toward and experiences with DiGA (Multimedia Appendix 1). To ensure that a vast variety of profiles and views on DiGA were represented, we used a purposive sampling approach to identify heterogeneous interviewees across various age groups, medical specializations, attitudes toward digitization, and geographic locations in Germany.

Interviews were conducted one-on-one by three independent researchers via video conference, telephone, or face-to-face. Interviews were conducted until all researchers agreed that further interviews were unlikely to surface major new viewpoints or topics. In total, 18 interviews with physicians and psychotherapists were conducted. These lasted between 25 and 60 minutes and covered four question categories: (1) attitudes toward DiGA, (2) prescription behavior and intentions, (3) barriers to DiGA prescription, and (4) potential remedies.

During each interview, interviewers wrote extensive notes. These were subsequently aggregated and reviewed by an expert panel consisting of five members with multi-professional backgrounds in medicine, natural sciences, and business and used for survey questionnaire design. In the first round of iteration, 38 survey questions were generated. These were prioritized in the second round of iteration, resulting in 25 questions. Next, answer options were developed based on the results from the qualitative interviews. Questions were also rephrased as Likert-scale items, most often with responses ranging from 1 (strongly disagree) to 5 (strongly agree).

### Online Survey

We next conducted a cross-sectional survey investigating health care professionals’ interactions with DiGA along four key categories discussed in the qualitative interviews. To establish a similar understanding of DiGA compared to general health and wellness apps among all survey respondents, an introductory information page about DiGA was displayed. We pretested the survey questionnaire with five colleagues and additional health care professionals to ensure survey comprehensibility and clarity. Question wording, survey functionality, and/or the introductory information page about DiGA were adjusted after each pretest, where necessary. The final questionnaire (Multimedia Appendix 2) was administered using Qualtrics, a web-based survey tool [28].

The survey was conducted over a 6-week period between December 2020 and January 2021 in accordance with the Checklist for Reporting Results of Internet E-Surveys (CHERRIES) guidelines [29]. A total of 19,196 German general practitioners, physicians, and psychotherapists were invited to participate in the anonymous online survey via their publicly accessible professional email addresses. To preserve privacy, respondents were not asked to provide any identifiable information. Additionally, we did not track which invited participants had started or completed the survey, limiting our ability to use reminders. To motivate participation, respondents could download a comprehensive, custom-made information package about DiGA for health care professionals after survey completion, addressing the various uncertainties and questions about DiGA that surfaced during our qualitative interviews and pretests. No financial incentive was offered.

In addition to insights from our qualitative interviews, we report findings from 17 out of 25 online survey questions asked. Besides descriptive analyses, dependencies between health care professional characteristics and attitudes toward DiGA as well as the likelihood of prescribing were tested in RStudio (version 1.3.1056) using chi-square tests or, when conditions for using chi-square tests were not met, Fisher exact tests with Monte Carlo approximation and 2000 replicates [30,31]. If respondents did not answer a particular question, they were excluded from the total number of respondents of this question in the analysis.

### Ethics Approval

This study was approved by the ethics committee of Witten/Herdecke University (reference No. 278/2020).

## Results

### Qualitative Interviews

Most respondents viewed DiGA positively. More flexible access to care independent of a practice’s opening hours and availability of therapy location, patient empowerment through increased sense of responsibility and self-efficacy, and improved adherence emerged as key potential benefits. While respondents had some experience with general mHealth apps, no respondent had prescribed DiGA so far. Some were generally open to doing so in the future. Yet, all respondents saw substantial barriers associated with prescribing DiGA, most importantly, lack of information, uncertainties regarding therapeutic benefits and medical evidence, and technical concerns. For some respondents, the low number of available DiGA relevant to their practice posed an additional barrier. All interviewees highlighted the desire to be informed more broadly. Some interviewees also called for stronger medical evidence and better compensation of services related to DiGA. These findings were further tested in the subsequent online survey.

### Online Survey

#### Demographics

A total of 1308 health care professionals completed the questionnaire, with minor nonresponse to individual questions, corresponding to a response rate of 7%, in line with previous research [23,32,33], making this the largest study on health care professionals’ mHealth adoption in Germany so far.

As shown in Table 1, the median age of respondents was 46 to 55 years, with 52.7% (682/1295) male and 47.2% (611/1295) female respondents, both representative of the overall German medical profession [34]. Most respondents hailed from urban areas (76.8%), predominately medium-sized cities between 20,000 and 100,000 inhabitants (406/1298, 31.3%), large cities between 100,000 and 500,000 inhabitants (304/1298, 23.4%), followed by small cities under 20,000 inhabitants (287/1298, 22.1%). A vast majority of respondents (1260/1296, 97.2%) were active in outpatient settings. About half of the respondents were active in single practices without physician and psychotherapeutic colleagues (613/1268, 48.3%), while the other half (655/1268, 51.7%) worked jointly with at least one colleague, a fact in line with doctors and psychotherapists in Germany overall [34]. Nearly all responding health care professionals participated in the German statutory health insurance scheme, although 93.6% (1171/1251) also accepted privately insured patients.

**Table 1:** Distribution of sociodemographic characteristics of all general practitioners, physicians, and psychotherapists (N=1308) who participated in the survey.

Characteristic		Respondents, n (%)
**Age in years (n=1295)**		
	<26	1 (0.1)
	26-35	49 (3.8)
	36-45	233 (18.0)
	46-55	415 (32.0)
	56-65	477 (36.8)
	>65	120 (9.3)
**Gender (n=1295)**		
	Male	682 (52.7)
	Female	611 (47.2)
	Diverse	2 (0.2)
**Practice location size: inhabitants (n=1298)**		
	<5000	85 (6.5)
	5001-20,000	287 (22.1)
	20,001-100,000	406 (31.3)
	100,001-500,000	304 (3.4)
	>500,000	216 (16.6)
**Practice type (n=1296)**		
	Hospital	28 (2.2)
	Single practice	613 (47.3)
	Joint practice	647 (49.9)
	Other occupation	8 (0.6)
**Practice size: practicing physicians or psychotherapists (n=1268)**		
	1	613 (48.3)
	2	270 (21.3)
	3	139 (11.0)
	4	101 (8.0)
	5	41 (3.2)
	6-10	64 (5.0)
	>10	40 (3.2)
**Patient population (n=1251)**		
	Statutory health insurance only	70 (5.6)
	Private health insurance only	10 (0.8)
	Both statutory and private health insurance	1171 (93.6)
**Medical specialty (n=1260)**		
	Anesthesiology	24 (1.9)
	Child and adolescent psychiatry and psychotherapy	61 (4.8)
	Dermatology	22 (1.7)
	Ear, nose, and throat medicine	38 (3.0)
	General medicine	284 (22.5)
	Gynecology	65 (5.2)
	Internal medicine	130 (10.3)
	Neurology	19 (1.5)
	Ophthalmology	18 (1.4)
	Orthopedics and trauma surgery	44 (3.5)
	Pediatrics	50 (4.0)
	Psychiatry and psychotherapy	65 (5.2)
	Psychological psychotherapy	264 (21.0)
	Psychosomatic medicine and psychotherapy	93 (7.4)
	Surgery	19 (1.5)
	Urology	19 (1.5)
	Other specialties	45 (3.6)

#### Perceived Benefits From and Attitudes Toward DiGA

A total of 62.1% (807/1299) of health care professionals viewed the fact that physicians can prescribe DiGA as positive or very positive. Only 22.6% (293/1299) viewed this recent development as negative or very negative in addition to 15.3% (199/1299) who viewed it neutrally. While health care professionals who had higher digital affinity (χ^2^_36_=126.7, *P*<.001) or were female (Fisher exact *P*=.01) held significantly more positive attitudes, the strength of the association between digital affinity, measured as self-rating for job-related digital competency or gender on the one hand and attitude towards DiGA on the other hand was rather weak (Cramer V=0.16 and 0.09, respectively). Medical specialty significantly influenced attitudes toward DiGA (Fisher exact *P*=.001; Cramer V=0.14). Other professional characteristics, such as age, practice type, size, and location and patient population, did not show significant effects on attitude.

**Figure 1:**
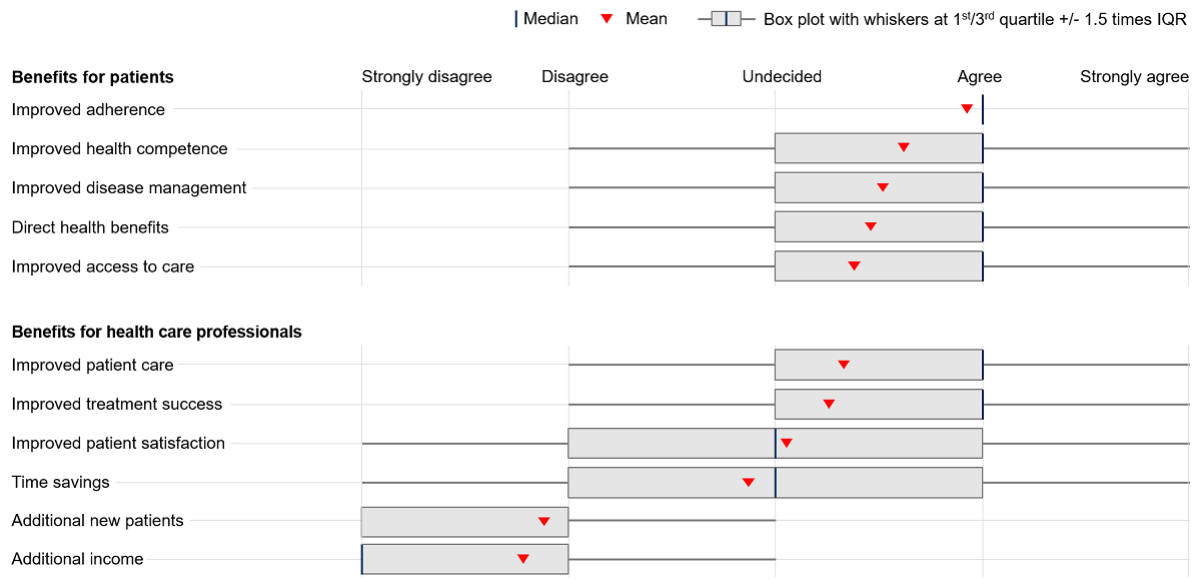
Perceived benefits from DiGA for patients and health care professionals. Respondents were asked to indicate the extent to which they see various benefits from DiGA on 5-point Likert scales. DiGA: Digitale Gesundheitsanwendungen (digital health applications).

Positive attitudes toward DiGA may be explained by the various benefits that health care professionals expect from DiGA for both patients and physicians: health care professionals who perceived greater benefits from DiGA held significantly more positive attitudes toward them (χ^2^_16_=116.5-785.3, *P*<.001; Fisher exact *P*<.001; Cramer V=0.12-0.42, depending on the individual benefit; see Figure 1 for respective benefits). On average, benefits for patients were considered to be larger than those for physicians, as shown in Figure 1. With 77.0% of respondents (997/1294), improved therapy adherence was identified as a benefit for patients most often, followed by increased health competence (842/1294, 65.0%), improved disease management (783/1294, 60.5%), direct health benefits from using DiGA (733/1295, 56.7%), and improved access to care (705/1294, 54.4%)*.* These benefits were seen to accrue primarily among younger patients. A total of 40.7% (527/1295) of health care professionals would prescribe DiGA primarily to younger patients.

About 1 in 2 health care professionals saw improved patient care (727/1287, 56.5%) as a benefit of DiGA for physicians. Increased patient satisfaction was seen as a benefit by 43.2% of respondents (556/1287), followed by time savings (410/1287, 31.9%). Acquiring new patients (82/128, 76.3%) and receiving additional income through reimbursement for medical services related to DiGA (26/128, 72.0%) were rarely seen as benefits. At the same time, one-fifth of health care professionals (234/1287, 18.2%) indicated that they were unable to assess whether DiGA would lead to attractive reimbursement, significantly more than for other potential physician benefits.

#### Prescription Intentions

A large majority of health care professionals have not prescribed DiGA and did not intend to do so in the next year: less than 10% (103/1299) of health care professionals indicated that they had prescribed DiGA. Only 30.3% (393/1299) of health care professionals planned to prescribe DiGA in the next 12 months. A total of 19.9% (259/1299) were uncertain as to whether they would prescribe DiGA and 49.8% (647/1299) did not plan to do so. Those who held more positive attitudes toward DiGA (χ^2^_16_=570.3, *P*<.001; Cramer V=0.33) or saw larger benefits from DiGA (χ^2^_16_=215.4-409.0, *P*<.001; Fisher exact *P*<.001; Cramer V=0.11-0.30, depending on the individual benefit) were believed to be significantly more likely to prescribe. Apart from digital affinity (χ^2^_36_=79.0, *P*<.001; Cramer V=0.12), health care professionals’ demographics were not significantly associated with prescription intentions.

**Figure 2:**
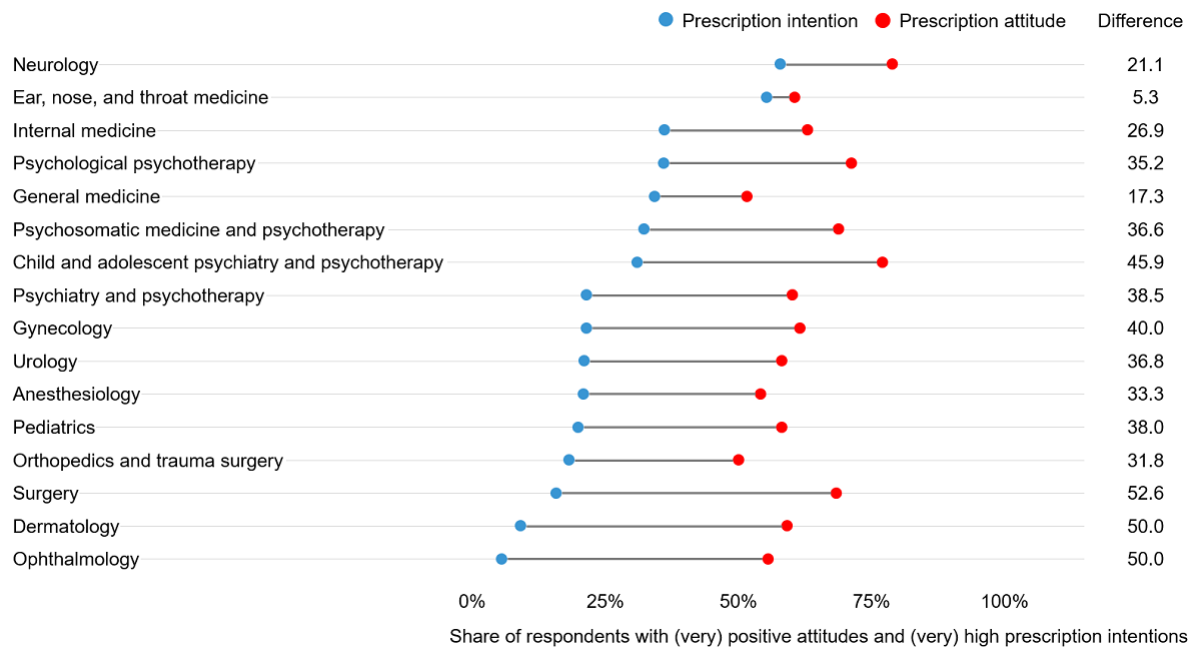
Prescription attitude and intention by medical specialty. Prescription attitude represents the share of respondents who expressed positive or very positive attitudes toward prescribing DiGA. Prescription intention represents the share of respondents who indicated that they would be likely or very likely to prescribe DiGA during the coming year. The difference shows the gap between prescription attitude and intention by medical specialty. See Table 1 for respective sample sizes per medical specialty. DiGA: Digitale Gesundheitsanwendungen (digital health applications).

Prescription intentions varied largely by medical specialty (Figure 2). Across all specialties, 30.3% (393/1299) of respondents indicated that they would be likely or very likely to prescribe DiGA in the coming year. Neurologists (11/19, 58%) and ear, nose, and throat doctors (21/38, 55%) held the highest prescription intentions. At the lower bound, only 6% (1/18) of professionals from ophthalmology intended to prescribe.

Similar but smaller variations across specialties were found for attitudes toward DiGA prescription. Across all specialties, 62.1% (807/1299) of respondents held positive or very positive attitudes. Neurologists held the most positive attitudes toward DiGA (15/19, 79%). At the other end of the spectrum, only 50% (22/44) of orthopedists and trauma surgeons did so.

On average, prescription intentions were more than 30 percentage points lower than prescription attitudes. This gap was smallest for ear, nose, and throat doctors (5.3 percentage points): 61% (23/38) of responding ear, nose, and throat professionals displayed high prescription attitudes and 55% (21/38) displayed an intention to prescribe. The gap was largest for surgeons: 68% (13/19) held positive prescription attitudes, yet only 16% (3/19) reported prescription intentions, with a gap of 52.6 percentage points. Despite this general trend, some of the results for prescription intentions, attitudes, and their relative gap may also be influenced by the comparatively small sample size in some medical specialties.

#### Perceived Barriers to Prescription

**Figure 3:**
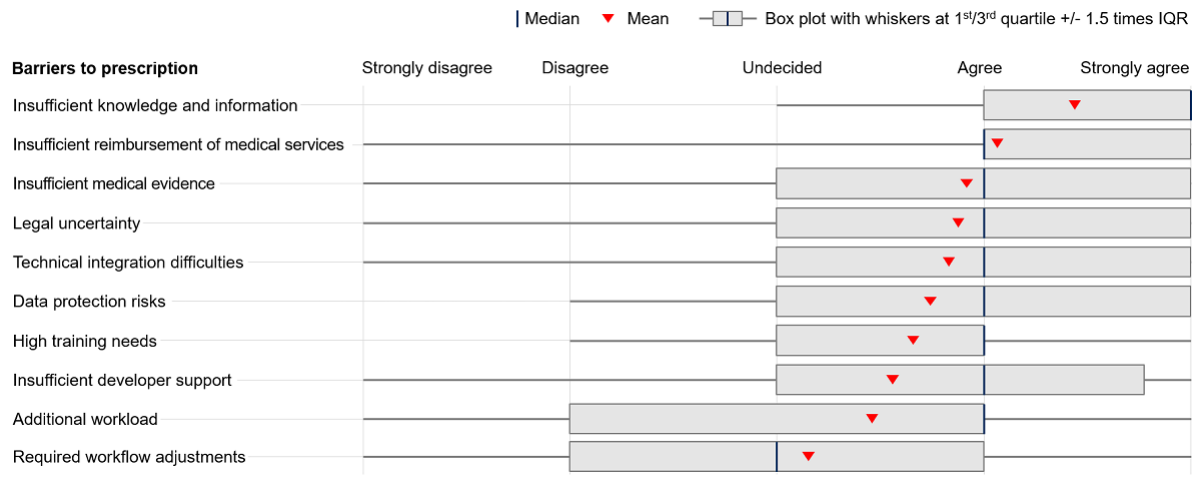
Perceived barriers to prescribing DiGA by health care professionals. Respondents were asked to indicate to what extent they believed various barriers prevented health care professionals from prescribing DiGA on 5-point Likert scales. DiGA: Digitale Gesundheitsanwendungen (digital health applications).

As Figure 3 displays, health care professionals saw significant barriers to prescribing DiGA across several dimensions. Above all, 87.4% (1135/1299) of health care professionals viewed insufficient information as an obstacle to DiGA prescriptions. This translates into low perceived competence in dealing with DiGA: about 7 out of 10 health care professionals felt insufficiently knowledgeable to differentiate bad from good DiGA (915/1298, 70.5%) and to advise patients regarding their application (905/1308, 69.2%). However, 92.4% (1208/1308) of health care professionals wanted to receive information about DiGA, thereby showing openness to address the key barrier to adoption of DiGA.

Additionally, a majority of health care professionals saw insufficient reimbursement of medical services related to DiGA (716/1299, 55.1%), insufficient evidence (712/1298, 54.9%), legal insecurities about potential liabilities for mistreatment (680/1299, 52.3%), and worries about data protection and security (658/1299, 50.7%) as clear barriers. Slightly less than half of the respondents believed that training needs for the respondent and potential staff (632/1299, 48.7%), perceptions of increased workload (584/1299, 45.0%), and technical integration issues (560/1299, 43.1%) were preventing health care professionals from adopting DiGA more broadly. Only about one-third of health care professionals saw workflow adjustment needs (431/1299, 33.2%) and missing support for health care professionals from DiGA providers (eg, for technical issues in daily operations; 372/1298, 28.7%) as obstacles to prescribing.

#### Measures to Support Adoption

**Figure 4:**
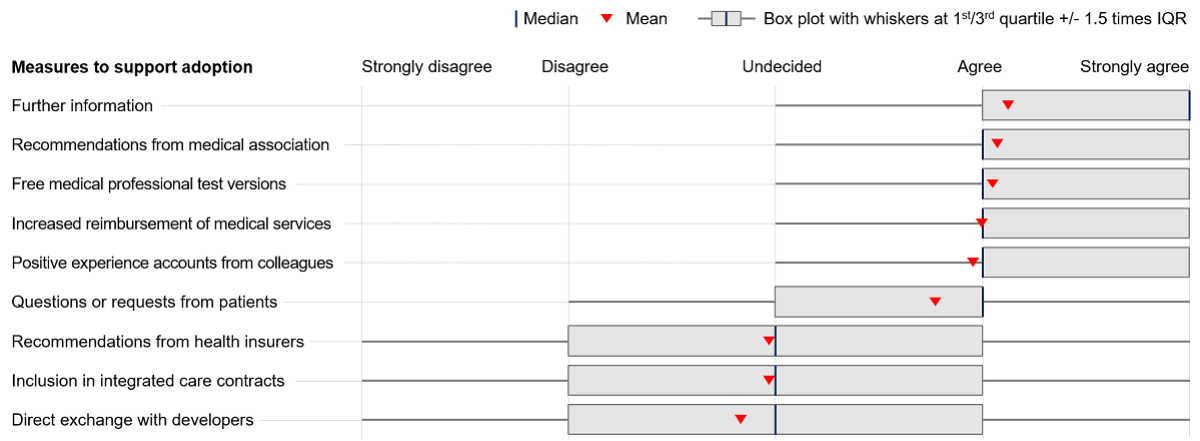
Measures to support health care professionals’ adoption of DiGA. Respondents were asked to indicate to what extent they believed various measures could help health care professionals to adopt DiGA on 5-point Likert scales. DiGA: Digitale Gesundheitsanwendungen (digital health applications).

Six measures were viewed positively by health care professionals to increase willingness to prescribe DiGA (Figure 4). Additional information about DiGA (1104/1297, 85.1%), recommendations by medical associations (1041/1297, 80.3%), positive experience reports about DiGA from medical colleagues (1024/1297, 79.0%), opportunities to test apps (1010/1297, 77.9%), and increased reimbursement for medical services related to DiGA (932/1297, 71.9%) have the potential to support health care professionals in the adoption of DiGA. When approached by patients, health care professionals also believed they would be more likely to engage with the topic and, thereafter, potentially prescribe DiGA (821/1297, 63.3%).

As displayed in Figure 4, other measures were viewed as neutral or ineffective. Recommendations to health care professionals by health insurers (529/1297, 40.8%), integrated care contracts (464/1296, 35.8%), and direct exchanges between health care professionals and developers (361/1297, 27.8%) were believed to have a weaker effect.

## Discussion

Despite the high potential of mHealth to improve medical care at lower costs [35,36], broad adoption has been challenging in the past. To overcome these challenges, Germany embarked on a new path by being the first country, worldwide, to introduce DiGA as prescribable mHealth apps into regular care in October 2020. However, DiGA adoption has been relatively slow, even at a time when large numbers of health care professionals have adopted telemedicine due to the COVID-19 pandemic [37]. To our knowledge, this study was the first to systematically examine the dynamics underlying the adoption of prescribable mHealth apps.

Our findings show that a majority of health care professionals support the introduction of DiGA into standard care, as they see significant medical benefits for patients, most importantly, improved patient adherence, health literacy, disease management, access to care, and direct health benefits. Although further research on the evidence of mHealth apps is needed in general [10], patient benefits have already been confirmed for various DiGA in randomized controlled trials [38-41].

Countless studies have found the expectation of benefits, positive attitudes, or perceived usefulness of mHealth technologies to be core predictors of adoption [20,21]. Accordingly, health care professionals are more likely to use a technology when they believe it to be beneficial to their patients’ care or themselves [9] and refrain from doing so when skeptical of its benefits for their practice [7].

While our findings confirmed a positive relationship between perceived usefulness and intention to use, the effect seems to be somewhat limited. Despite the multitude of benefits of DiGA seen by our respondents, only about one-third of health care professionals planned to prescribe DiGA in the future. Although this finding is in line with mHealth adoption rates in other countries [32], the share of health care professionals who have already prescribed DiGA is drastically smaller in Germany, seconding the need for further investigations of relevant factors.

While some studies consider gender and age as sociodemographic factors influencing technology adoption [21], others find this effect to be limited to attitude, not intention to prescribe [7]. The latter is true for our survey results. Only digital affinity had a significant and positive effect on both attitude and prescription intention. This may be due to the fact that health care professionals with greater digital affinity and information and communications technology experience anticipate greater ease of use when integrating DiGA into their work, a factor that has been found to be a strong predictor of technology adoption [9,42].

In addition to the potential effects of sociodemographics, two other factors may explain the low prescription intentions of DiGA. First, the availability of relevant DiGA is limited for some specialties, which may, therefore, result in these health care professionals not planning to prescribe DiGA, a factor also highlighted by our qualitative interviews. Looking at the 20 apps that have been approved so far, 10 of them are related to psychotherapy (eg, depression, phobias, and insomnia), 4 are related to neurology (eg, stroke, multiple sclerosis, and migraine), and 1 is related to nutrition (ie, obesity) [43]. These are largely irrelevant for professionals from specialties with the largest gap between positive attitudes and prescription intentions (ie, ophthalmology, dermatology, surgery, and other specialties). However, medically beneficial mHealth apps targeting diseases in these currently underrepresented specialties (eg, smartphone-based early detection of skin cancer [44] or treatment of ophthalmologic conditions, such as amblyopia and glaucoma [45]) are starting to emerge or are already under review by the German Federal Institute for Drugs and Medical Devices [46] and may increase DiGA prescriptions in the future.

Second, barriers to adoption may explain low prescription intentions. Barriers identified in our study include lack of information and medical evidence; insufficient reimbursement of medical services; concerns about medico-legal issues, such as liability and data protection risks; as well as workflow-related issues, including required workflow adjustments, training needs, and increased workloads. Most of these barriers are consistent with those identified by other studies from various countries and settings. A recent systematic review by Jacob et al [7] identified workflow-related factors; privacy, security, and medico-legal concerns; and monetary issues related to reimbursement and fees to be among the most studied and important social and organizational factors that influence technology adoption by health care professionals. Interestingly, lack of information—with over 87% of responses reporting this as the largest barrier for adoption in this study—has been studied significantly less [7]. This may be because past research has frequently studied conceptually more established and mature concepts, such as electronic health records [15], contrary to Germany’s DiGA, which had only been available for under 3 months at the time of this study. For such novel technology, information may be an anteceding barrier that needs to be addressed first before health care professionals become fully aware of more frequently studied barriers to adoption.

To address these barriers and support adoption of DiGA, five concrete measures should be implemented. First, increasing health care professionals’ level of information and trust in DiGA through recommendations from reliable bodies, such as medical associations, scientific societies, opinion leaders, and peers [7,24,47], and enabling health care professionals to experience DiGA themselves through free test versions may foster adoption. Here, it is critical to address the barriers perceived, such as medico-legal concerns around liability for mistreatment and data risks, as well as benefits from using DiGA for both patients and health care professionals. Second, introducing DiGA-related medical services into the remuneration system for statutory health insurance–accredited health care professionals may offer stronger financial incentives for adoption. Past research from Germany suggests that such measures may influence up to 85% of health care professionals in adopting a new technology [25].

Third, scientists should further investigate medical evidence of DiGA using robust study designs (eg, randomized controlled trials and meta-reviews according to Cochrane standards) and make findings freely available more than is currently the case. Moreover, given the widespread lack of awareness, previous results should be disseminated more effectively, starting with the national DiGA directory operated by the German Federal Institute for Drugs and Medical Devices. A more transparent, standardized, and, thus, more accessible presentation of evidence, with a clear indication of medical and structural effects for patients and study design conditions complied with, may promote trust in DiGA [48]. Fourth, training offerings related to DiGA should be expanded to help physicians make decisions about DiGA implementation within their own work at an extensive and intensive margin. Providing incentives for trainings, for instance, continuing medical education certification, may further aid this effort. Fifth, ensuring compatibility of DiGA with existing clinical practices, workflows, and infrastructure will be critical to remove barriers to adoption [15].

This study extends our understanding of the dynamics underlying the adoption of prescribable mHealth apps by health care professionals. Given that an online survey was used, our results may be subject to some self-selection bias and, therefore, bounded representativeness. Further research may, therefore, wish to validate these findings with an even larger, more representative sample.

In conclusion, three strands of research resulted from this study. First, given the criticality of greater information for prescription among medical professionals, future studies should investigate which channels appear to be most appropriate for delivering DiGA information and which types of content are most critical for health care professionals. Second, to reduce reliance on health care professionals who might remain reluctant to prescribe DiGA, other paths to support the adoption of medically beneficial DiGA should be explored. Third, further research should investigate whether health care professionals are reluctant to prescribe DiGA to some patient groups (eg, those lacking language or digital skills) and how such potential digital divides can be addressed to realize mHealth’s full potential for patients and in the German health care system at large.

## Appendix

Multimedia Appendix 1 Translated interview guide.

Multimedia Appendix 2 Translated survey questionnaire.

## Abbreviations

BfArM: Bundesamt für Arzneimittel und Medizinprodukte (German Federal Institute for Drugs and Medical Devices)
CHERRIES: Checklist for Reporting Results of Internet E-Surveys
DiGA: Digitale Gesundheitsanwendungen (digital health applications)
mHealth: mobile health
