# Supplementary material for: Physicians’ Attitudes Toward Prescribable mHealth Apps and Implications for Adoption in Germany: Mixed Methods Study
Source: JMIR Mhealth Uhealth. 2021 Nov 23;9(11):e33012. doi: 10.2196/33012 (PMC8663495; doi:10.2196/33012)
Supplement: Multimedia Appendix 1 [file mhealth_v9i11e33012_app1.pdf]

Interview guideline for qualitative interviews with healthcare professionals

Introduction of interviewer and general research area

Introduction to interview format

Information about anonymity, data protection, and data processing

1. Could you tell me a bit about yourself, including your professional background and current role?
2. Have you heard of the terms “DiGA”, “Digitale Gesundheitsanwendung” or “App auf Rezept”?

Brief introduction to DiGA and corresponding legislation

3. What do you think about the fact that doctors are now allowed to prescribe DiGA?

4. In your view, what are the biggest chances or advantages of DiGA?
5. Do you expect that DiGA will benefit patients? How so?
6. Do you expect that DiGA will benefit healthcare professionals? How so?
7. In your view, what are the biggest risks or disadvantages of DiGA?

8. Have you prescribed one or more DiGA so far?
9. What are the reasons for this?

10. Do you consider prescribing DiGA in the future?
11. What are the reasons for this?

12. What do you believe are the main barriers that people like you do not prescribe DiGA?
13. How difficult do you think it will be to prescribe, integrate, and use DiGA in practice?
14. How could these barriers be addressed?
