# Supplementary material for: Physicians’ Attitudes Toward Prescribable mHealth Apps and Implications for Adoption in Germany: Mixed Methods Study
Source: JMIR Mhealth Uhealth. 2021 Nov 23;9(11):e33012. doi: 10.2196/33012 (PMC8663495; doi:10.2196/33012)
Supplement: Multimedia Appendix 2 [file mhealth_v9i11e33012_app2.pdf]

Survey questionnaire for healthcare professionals
(The questionnaire was presented to respondents in German. This copy was automatically translated, only stating questions included in the publication.)

1. Introduction page:

Which DiGAs are available? How do DiGA work? What benefits do DiGA offer to whom? How do I prescribe DiGA? How do my patients obtain and use a DiGA and how do I support them?

Answers to these and other questions can be found in the information folder "DiGA for your practice: What I need to know now", which you can download free of charge after completing the following survey by Witten/Herdecke University. In a brief and clear manner, the folder contains the most important information about DiGA for your practice.

As part of doctoral research at the Faculty of Health, this research investigates the attitudes of psychotherapists and doctors of all specialties towards digital health applications (DiGA), including those who have had little or no previous contact with digital health applications. The goal is to help practitioners deal with this new form of care. The survey will take 4-7 minutes to complete.

Thank you for your participation and contribution to the research!

2. Participant consent

This survey is anonymous and does not reveal your identity. All survey data is solely used for the purpose of scientific research. Participation is voluntary. Before proceeding, you are requested to read the information on data processing in the context of the study.

By clicking the button below, you acknowledge that you have read the attached information and consent.

I have read and agree to the information on the processing of survey data.

3. Information page: How are DiGA different from health apps?

What are DiGA?
DiGA are digital applications for patients that help detect, monitor, and treat or alleviate disease.

What distinguishes DiGA from other health apps?
DiGA are approved as medical devices and additionally licensed by the Federal Institute for Drugs and Medical Devices (BfArM). DiGA can be prescribed by doctors and psychotherapists and are reimbursed by all statutory health insurers - they are therefore also called "apps on prescription".

Which DiGA are available?
So far, 6 DiGA have been approved for the treatment of tinnitus ("kalmeda"), anxiety disorders ("velibra" and "invirto"), obesity ("zanadio"), as well as "somnio" for sleep training and "vivira" for exercise therapy. Various other applications in a wide range of indications are currently in the approval process.

We ask you to complete this questionnaire only with regard to DiGA ("apps on prescription") and not health apps in general.

4. Survey questions on DiGA

How much do you agree with the following statement? "I am in favor of doctors and therapists being able to prescribe digital health applications ("apps on prescription")."

- ○ Strongly disagree
- ○ Disagree
- ○ Undecided
- ○ Agree
- ○ Strongly agree

How much do you agree with the following statement? "I feel sufficiently informed about digital health applications ("apps on prescription") to prescribe them."

- ○ Strongly disagree
- ○ Disagree
- ○ Undecided
- ○ Agree
- ○ Strongly agree

How do you rate your competence when it comes to …

	Very bad	Bad	Neither bad nor good	Good	Very good
... surveying the range of available digital health applications?	○	○	○	○	○
... advising patients on digital health applications?	○	○	○	○	○
... distinguishing good from bad digital health applications?	○	○	○	○	○

How often are you asked by your patients about digital health applications ("apps on prescription") in general or about their prescription?

- ○ Never
- ○ Less than once per month
- ○ Monthly
- ○ Weekly
- ○ Daily

How much do you agree with the following statement? "My patients expect me to prescribe digital health applications ("apps on prescription")."

- ○ Strongly disagree
- ○ Disagree
- ○ Undecided
- ○ Agree
- ○ Strongly agree

How often do you prescribe digital health applications ("apps on prescription")?

- Never
- Less than once per month
- Monthly
- Weekly
- Daily

How likely do you think you are to prescribe digital health applications ("apps on prescription") in the next 12 months?

- Very unlikely
- Rather unlikely
- Undecided
- Rather likely
- Very likely

How much do you agree with the following statement? "I am more likely to prescribe digital health applications ("apps on prescription") to young patients."

- Strongly disagree
- Disagree
- Undecided
- Agree
- Strongly agree

In your opinion, which aspects represent the greatest benefits of digital health applications ("apps on prescription") for patients?

	Strongly disagree	Disagree	Undecided	Agree	Strongly agree	I don’t know
Direct health benefits, e.g., additional treatment options or more guideline-based therapy	○	○	○	○	○	○
Improved disease management, e.g., better coordination of treatment processes or better management of disease-related difficulties in everyday life	○	○	○	○	○	○
Improved adherence, e.g., reminders of agreed therapy measures in everyday life and motivation to adopt health-conscious behavior	○	○	○	○	○	○
Improved access to care, e.g., bridging therapy wait times, more comprehensive or lower-threshold care options	○	○	○	○	○	○
Improved health literacy and competence, e.g., through better education of patients	○	○	○	○	○	○

Other benefits of digital health applications for patients (optional)

Free text

In your opinion, which aspects represent the greatest advantages of digital health applications ("apps on prescription") for healthcare professionals?

	Strongly disagree	Disagree	Undecided	Agree	Strongly agree	I don’t know
Acquisition of new patients	○	○	○	○	○	○
Greater patient satisfaction	○	○	○	○	○	○
Long-term time savings per patient through efficiency gains	○	○	○	○	○	○
Improved patient care	○	○	○	○	○	○
Improved treatment success	○	○	○	○	○	○
Additional income	○	○	○	○	○	○

Other benefits of digital health applications for healthcare professionals (optional)

Free text

What do you see as the biggest barriers to prescribing digital health apps?

	Strongly disagree	Disagree	Undecided	Agree	Strongly agree	I don’t know
Lack of information, e.g., on available applications, suitability of individual DiGA, and/or prescribing and reimbursement process	○	○	○	○	○	○
Lack of or insufficient evidence of benefit for patients	○	○	○	○	○	○
High initial training and/or familiarization effort for medical personnel	○	○	○	○	○	○
Necessary adjustments and adaptations of existing treatment or practice processes	○	○	○	○	○	○
Poor integration or compatibility with existing practice software and tools	○	○	○	○	○	○
Lack of support from the manufacturer for technical problems or questions	○	○	○	○	○	○
Permanently increased workload of doctors/therapists due to additional tasks in the care of patients	○	○	○	○	○	○
Inadequate reimbursement of doctoral/therapeutic concomitant services, e.g., monitoring patient data and responding to follow-up questions	○	○	○	○	○	○
Uncertainty about privacy and security of personal (health) data	○	○	○	○	○	○
Other legal ambiguities, e.g., regarding liability risks in the event of misdiagnosis or treatment based on data from DiGA	○	○	○	○	○	○

Other barriers to prescribing digital health applications (optional)

Free text

What might encourage you to prescribe (more) digital health apps?

	Strongly disagree	Disagree	Undecided	Agree	Strongly agree	I don't know
More information on available applications, suitability of individual DiGA, and/or prescribing and reimbursement process	○	○	○	○	○	○
Higher reimbursement for concomitant services, e.g., through EBM code, lump sum or extrabudgetary payments	○	○	○	○	○	○
Opportunity to try out digital health applications for yourself as part of a trial version	○	○	○	○	○	○
Recommendation of individual DiGA by health insurers	○	○	○	○	○	○
Integration of DiGA into integrated care contracts/selective contracts	○	○	○	○	○	○
Questions or requests by own patients	○	○	○	○	○	○
Direct exchange with the manufacturer of a digital health application	○	○	○	○	○	○
Positive experience reports from colleagues, e.g., at medical congresses or in professional journals	○	○	○	○	○	○
Recommendation of one or more DiGA by scientific medical societies/guidelines	○	○	○	○	○	○

Other factors that may encourage you to prescribe digital health applications (optional)

Free text

Which information on digital health applications are you interested in? (Multiple selection possible)

- What are the applications?
- For which patients and indications is a DiGA suitable?
- What are the proven benefits and risks of DiGA?
- What does a DiGA cost and which health insurance companies reimburse it and how?
- How do I prescribe a DiGA?
- How do my patients get a DiGA after I prescribe it, and how do they use it?
- How do I monitor the use of DiGA?
- Other (please specify)
- I do not wish to receive any additional information about DiGA.

Here you have the opportunity to explain your view on digital health applications in more detail (optional).

Free text

5. Demographic questions

What is your medical specialization?

Dropdown

Where do you work as a doctor or therapist? In a city/town with...

- more than 500,000 inhabitants
- 100,001 to 500,000 inhabitants
- 20,001 to 100,000 inhabitants
- 5,001 to 20,000 inhabitants
- less than 5,000 inhabitants

Which type of work location best describes your job situation?

- Clinic
- Individual practice (without other colleagues)
- Practice with other colleagues (e.g., joint practice or group practice)
- Other

Are you a statutory-health insurance licensed doctor or a private practitioner?

- Only statutory-health insurance licensed
- Only private practitioner
- Both

How many doctors or therapists work in your practice?

Free text

How digitally affine do you consider yourself to be in a professional context?

Scale 1 (not at all digitally affine) to 10 (very digitally affine)

How old are you

- up to 25 years
- 26 to 35 years
- 36 to 45 years
- 46 to 55 years
- 56 to 65 years
- over 65 years

What is your gender?

- Male
- Female
- Divers
